# Time Interaction With Two Spatial Dimensions: From Left/Right to Near/Far

**DOI:** 10.3389/fnhum.2021.796799

**Published:** 2022-01-18

**Authors:** Michela Candini, Mariano D’Angelo, Francesca Frassinetti

**Affiliations:** ^1^Department of Psychology, University of Bologna, Bologna, Italy; ^2^Unit of Recovery and Functional Rehabilitation, Istituti Clinici Scientifici Maugeri IRCCS, Institute of Castel Goffredo, Mantova, Italy

**Keywords:** time perception, Muller-Lyer illusion, tool-use, near/far space, time bisection task

## Abstract

In this study, we explored the time and space relationship according to two different spatial codings, namely, the left/right extension and the reachability of stimulus along a near/far dimension. Four experiments were carried out in which healthy participants performed the time and spatial bisection tasks in near/far space, before and after short or long tool-use training. Stimuli were prebisected horizontal lines of different temporal durations in which the midpoint was manipulated according to the Muller-Lyer illusion. The perceptual illusory effects emerged in spatial but not temporal judgments. We revealed that temporal and spatial representations dynamically change according to the action potentialities of an individual: temporal duration was perceived as shorter and the perceived line’s midpoint was shifted to the left in far than in near space. Crucially, this dissociation disappeared following a *long* but not *short* tool-use training. Finally, we observed age-related differences in spatial attention which may be crucial in building the memory temporal standard to categorize durations.

## Introduction

In everyday activities, we commonly experience an intimate coupling between time and space. In this respect, it was largely demonstrated that the stimulus spatial position, on the left or on the right side, influences time perception (see Bonato et al., 2012 for a review). More recently, it was demonstrated that also the stimulus position in the depth dimension, near and far space, impacts time perception (Anelli et al., 2015). As far as the influence of *left/right spatial coding* on time estimation, it was demonstrated that the duration of stimuli presented on the left is perceived shorter as compared to stimuli presented on the right (Vallesi et al., 2008; Vicario et al., 2008; Frassinetti et al., 2009). These findings have contributed to formulating the hypothesis that humans represent time along a horizontal line (mental timeline) where temporal intervals are typically represented in ascending order from left to right (Santiago et al., 2007, 2010; Ishihara et al., 2008; Müller and Schwarz, 2008; Weger and Pratt, 2008; Magnani et al., 2011; Vallesi et al., 2011; Di Bono et al., 2012; Oliveri et al., 2013; Isham et al., 2018).

*Near/far spatial coding* influences time perception too: stimuli presented in far space are perceived shorter in duration as compared to stimuli presented in near space (Anelli et al., 2015). The distinction between near and far space depends on the extent to which an action can be performed (Rizzolatti et al., 1997; Berti and Frassinetti, 2000; Cardinali et al., 2009). Thus, near space is defined as the reachable space inside the arm’s reaching distance, and far space is defined as the unreachable space outside the reaching distance (Maravita and Iriki, 2004; Bartolo et al., 2014; De Vignemont and Iannetti, 2015).

Further evidence supporting the role of performing action *per se* or action capability of an individual in mediating the distinction between near and far space is provided by the effects of tool-use which extends action capabilities (Witt, 2011; Patané et al., 2016b; Biggio et al., 2017; Candini et al., 2019; Forsberg et al., 2019; see, for reviews, Johnson-Frey, 2004; Maravita and Iriki, 2004; Reynaud et al., 2016). In fact, tool-use allows to reach objects located in a far/unreachable space and, consequently, extends the reaching of boundary space (Berti and Frassinetti, 2000; Longo and Lourenco, 2006; Hunley et al., 2017; but see also D’Angelo et al., 2018; see, for review, Brozzoli et al., 2014; Martel et al., 2016).

The distinction between near and far space and its plasticity has widely been demonstrated in several behavioral studies adopting visuospatial tasks, such as the line bisection and landmark task, which require to identify the subjective midpoint of a horizontal line (Bisiach et al., 1998; Lindbergh and Kieffaber, 2013; Patané et al., 2016a). In these tasks, when stimuli are presented in near space, healthy participants typically exhibit a leftward bias that gradually shifts rightward as the stimulus is moved further away (Bowers and Heilman, 1980; Jewell and McCourt, 2000; Longo and Lourenco, 2006, 2007). However, some studies adopting a line bisection task reported the opposite, which is a rightward bias in near space compared to far space (Cowie and Hamil, 1998; Braun and Kirk, 1999; McCourt and Garlinghouse, 2000; Varnava et al., 2002). What is of interest here is that when using a tool, that extends the near/reachable space up to the far/unreachable space (i.e., a long tool), a bias similar to that observed in near space emerged also in far space (Longo and Lourenco, 2006). This is not true when a short tool is used that does not extend the action capability and does not allow to reach the far space (Farnè et al., 2005; Costantini et al., 2011; Bourgeois et al., 2014; Anelli et al., 2015; Patané et al., 2016b; Candini et al., 2019).

Furthermore, a relevant issue is that age influences the bias found in the line bisection task. However, the nature of this effect is somewhat controversial: some studies have demonstrated an attenuated bias with age (Schmitz and Peigneux, 2011; Schmitz et al., 2013; Benwell et al., 2014; Learmonth et al., 2015; Friedrich et al., 2018), whereas other studies have put in evidence an accentuated bias with age (Harvey et al., 1995, 2000) or no effects of age (Learmonth et al., 2017). In a similar vein, a still debated question concerns the role of age in time estimation: temporal processing appears to be altered in healthy aging which is often associated with a faster passage of subjective time (Craik and Hay, 1999; Perbal et al., 2002; Rueda and Schmitter-Edgecombe, 2009; Turgeon et al., 2016; Paraskevoudi et al., 2018). For instance, Craik and Hay (1999) found that older participants perceived stimuli duration as shorter, compared to the veridical temporal duration than younger participants. However, other studies found the opposite tendency in aging, which is to perceive longer stimuli duration compared to the veridical interval (Block et al., 1998; Turgeon et al., 2011). Thus, it remains unknown how aging actually influences and determines the response bias observed either in spatial or temporal judgments.

In this study, we concurrently investigated the relationship between time and two different spatial codings, namely, the horizontal extension along a transversal axis (from left to right) and the depth dimension along a sagittal axis (from near/reachable to far/unreachable). Moreover, according to several studies demonstrating that age influences *different abilities* (Sardone et al., 2020), such as spatial and time perception, we expect a different performance between young and elderly participants in temporal estimation as a function of the spatial coding of stimulus. To these aims, we study the effects on time perception of a visual illusion, altering the left/right spatial coding (Muller-Lyer illusion), and of the tool-use, modifying the boundary between near and far space.

Young and aging healthy participants were involved in two time experiments and two spatial experiments. In the time bisection task, participants estimated whether stimuli were “short” or “long” in duration, with respect to a previously acquired pair of references (1,600 and 2,400 ms). Stimuli consisted of horizontal prebisected lines and were displayed according to the Brentano version of the Muller-Lyer illusion. This type of illusion uses three different arrows arranged in such a way that one half of the line is apparently expanded, whereas the other half appears to be compressed (Coren and Girgus, 1978; Weidner and Fink, 2007). We predicted that if the spatial bias induced by the Brentano version of the Muller-Lyer illusion influences time perception when the transector is perceived as nearer to the left end of the line, time should be perceived as shorter, and when the transector is perceived as nearer to the right end of the line, time should be perceived as longer. Furthermore, the effects of depth on time and its plasticity were assessed by presenting stimuli both in near and far spaces, before and after *long tool-use training*. According to previous findings, time is expected to be perceived as shorter in the far compared to near space before but not after long tool-use training (Anelli et al., 2015; Petrizzo et al., 2021).

To verify the presence of illusory effects induced by the Muller-Lyer configuration, participants were involved in the spatial bisection task, in which the same stimuli of the time bisection task were used, except for stimulus duration which was not manipulated and was set at 2,000 ms. Participants were required to judge which end of the prebisected line the transector was closer to. Furthermore, two additional control experiments were also conducted in which the same time bisection task and spatial bisection task previously described were performed before and after a *short tool-use training* (Farnè et al., 2005; Costantini et al., 2011; Bourgeois et al., 2014; Anelli et al., 2015; Patané et al., 2016b; Candini et al., 2019).

We expect that testing the effect of Muller-Lyer illusion on time estimation will provide a critical contribution to understanding how visual perception influences time processing. Moreover, changes in time estimation might be driven by the possibility to act in space, regardless of the perceived illusory spatial bias. If that is the case, we should find that the body proximity and the active long tool-use training but not short tool-use training are effective to modulate time estimation.

## Experiment 1: Time Bisection Task

### Participants

A total of 36 right-handed neurologically healthy volunteers with normal or corrected-to-normal vision were recruited. The sample size was calculated by using G*Power 3.1.7, hypothesizing an estimated effect size of f = 0.4 and a power at 80% (Anelli et al., 2015). Notably, 18 volunteers were elderly participants (6 men, mean ± SD age = 57.9 ± 6.9 years; mean ± SD education = 13 ± 4.6 years), and the remaining 18 volunteers were younger participants (5 men, mean ± SD age = 23.4 ± 3 years; mean ± SD education = 17 ± 1.7 years).

All participants were naive to the purpose of the research and provided written informed consent to participate in the study. The study was approved by the local ethics committee (Department of Psychology, University of Bologna), and all procedures were in accordance with the ethical standards of the 2008 Declaration of Helsinki.

### Stimuli

**FIGURE 1:**
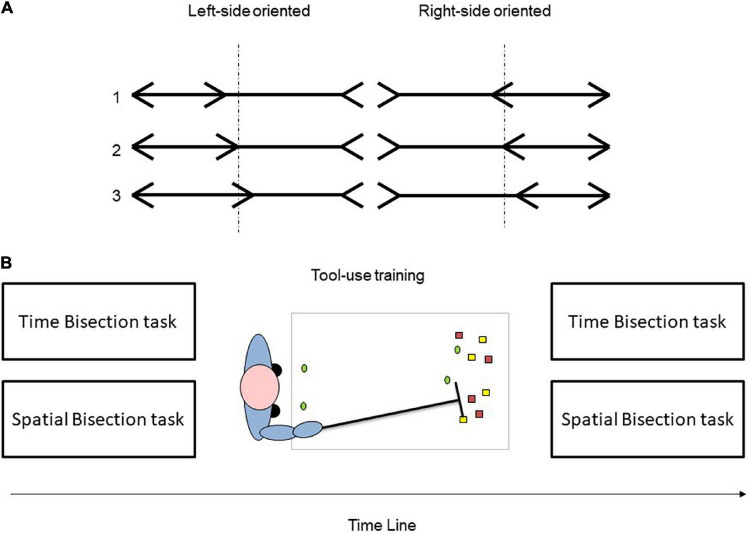
Stimuli and procedure adopted in the experimental tasks. In the upper panel, **(A)** experimental stimuli were depicted: stimuli varied according to the left/right side orientation, and the transector positions (1: −0.2°; 2: 0.0°; 3: + 0.2°). The vertical dot lines represent the true center of each prebisected line. In the lower panel, **(B)** a schematic representation of the procedure adopted was represented. Both tasks were performed before and after a tool-use training session in which the participant (blue) reached a series of colored chips placed by the experimenter outside the reaching space by using a rake.

Stimuli were black prebisected horizontal lines displayed on a white screen positioned at 60 cm (near space) or 120 cm (far space) from the eyes of participants. To ensure that the retinal angle remained constant [0.48° × 5.25° visual angle (VA)] across viewing distances, the size of stimuli was adjusted near space: 0.5 cm height and 5.5 cm length; far space: 1 cm height and 11 cm length). This phenomenon can be ascribed to the size constancy, a perceptual mechanism that enables us to perceive an object of different sizes presented at different viewing distances as having the same size. Indeed, our brain combines retinal images and distance cues to perceive objects as having the same physical properties, i.e., a constant size, despite changes in retinal input (Sperandio and Chouinard, 2015). Each horizontal line was bisected by a black arrow (transector) and two more black arrows were presented at the line’s ends, which could be oriented to the left or to the right side. Indeed, two different illusory conditions were presented: *Left expanded* (left-sided outgoing fin/right-sided ingoing fin) and *Right expanded* (left-sided ingoing fin/right-sided outgoing fin). Throughout this study, the two types of illusory stimuli are distinguished with reference to the orientation of outgoing fins (left or right expanded side). The fin formed with the line in 45° (ingoing; > <) or 315° (outgoing; < >) angle. Across trials, the stimuli varied according to (1) the 2 stimuli orientations (left/right), (2) the 3 positions at which the horizontal line was bisected (shift to the left: –0.2°; true center: 0.0° shift to the right: + 0.2°), and (3) the 5 stimuli durations (i.e., 1,600, 1,800, 2,000, 2,200, or 2,400 ms; Figure 1) for a total of 30 stimuli combinations. Each stimulus was repeated six times, thus obtaining a total of 360 trials. To reduce the effect of fatigue on the performance of participants, three different blocks of 60 trials were presented for each condition, i.e., 180 in the near space and 180 in the far space.

**FIGURE 2:**
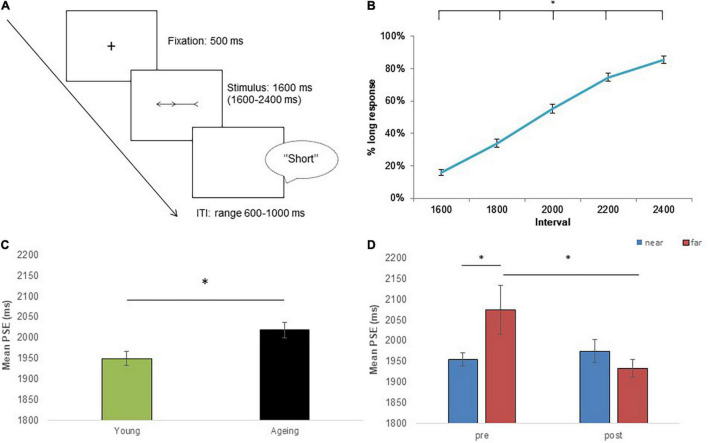
The experimental paradigm and performance of participants at time bisection task. In the time bisection task, **(A)** participants were asked to judge whether the duration of each stimulus was “short” or “long” with respect to pair of short (1,600 ms) and long (2,400 ms) reference durations. **(B)** The percentage of long response as a function of duration interval. **(C)** The point of subjective equality (PSE) judgments expressed as a function of Group. **(D)** The PSE is expressed as a function of tool-use session (pre and post) and conditions (near and far). Error bars indicate SEMs. Asterisks indicate significant differences.

Each trial began with the presentation of a fixation cross displayed on a white background for 500 ms, after which the stimulus was displayed for one of the five temporal durations (i.e., 1,600, 1,800, 2,000, 2,200, and 2,400 ms) and then replaced by a white background which remained visible until a verbal response was made. After the response, the subsequent trial was presented following a random inter-trial interval (range 600–1,000 ms; as shown in Figure 2A). Stimuli were centrally displayed on a 15” LCD monitor (resolution 1,366 × 768 pixels) and were presented by using the E-Prime 2.0 software package (Psychology Software Tool^©^ 1996–2012, United States).

### Procedure

Participants were comfortably seated directly in front of the monitor screen and were asked to rest their hands on their thighs. The experimenter ensured that the body position of the subject remained constant during the whole experiment.

The time bisection task consisted of the verbal classification of a series of prebisected lines that were displayed for different durations at the center of the computer screen. We adopted two-alternative forced-choice paradigms in which participants have to judge whether the duration of each line was “short” or “long” with respect to previously acquired pair of reference durations (1,600 and 2,400 ms). In the practice session, a total of eight trials were randomly presented, and participants had to classify four intervals of 1,600 ms as “short” and four intervals of 2,400 ms as “long.” Feedback was given on accuracy only for these eight practice trials. All participants reached at least 80% of accuracy with no more than two practice sessions (mean ± SD accuracy = 89.7% ± SD 0.09). Then, they were involved in the experimental task in which all the possible prebisected lines were randomly displayed. Participants were instructed to make their best guess if they were unsure and to respond as accurately and as quickly as possible. No feedback was given in the experimental blocks.

The experimenter seated behind the participants (at least 1.5 m) and recorded their verbal responses by pressing one of the two keys on a wireless mouse (left for “short”/right for “long”). Each block of the time bisection task took approximately 6 min to complete.

The time bisection task was performed in two experimental conditions: in near space (60 cm from the computer screen) and in far space (120 cm from the computer screen), presented in separate blocks, counterbalanced across participants. The task was repeated two times, in the same order, before and after a tool-use session.

### Tool-Use Session

During the tool-use training, the participant stood at the short side of a rectangular table. The experimenter randomly placed on the table a series of colored poker chips (red, green, yellow, white, and black) outside the reaching space, at a distance of ≈100 cm from the trunk of a participant. The chips were presented in the midsagittal axis (0°) of participants, or at 10° and 20° to the left and to the right of the central position (–10°, –20°, + 10°, and +20°; as shown in Figure 1). Each participant performed about a total of 100 reaching movements in order to bring the objects close to their body with no time constraint. During the tool-use session, participants had to use a 70-cm-long rake with their right hand. The tool-use training lasted for 15 min.

## Data Analysis

In our paradigm, the bisection point is the estimated temporal value (expressed in milliseconds) for which participants would respond “long” or “short” with equal probability. To estimate the bisection point, we first computed the percentage of “long” responses for each interval duration (1,600, 1,800, 2,000, 2,200, and 2,400 ms), and then cumulative Gaussian functions were fitted to the percentage of “long” responses across different stimulus durations using logistic regression. The point at which the psychometric function cuts 50% of “long” responses indicates the duration at which a participant is equally likely to classify the stimuli as short or long [point of subjective equality (PSE)]. For each participant, the PSE was separately calculated for each type of illusion (left/right orientation) and transector position (–0.2°; 0.0°; +0.2°) in each of the four conditions (i.e., pre-tool-use/near space, pre-tool-use/far space, post-tool-use/near space, and post-tool-use/far space). Bisection points below 2,000 ms reflect duration overestimation (i.e., durations are perceived longer than they actually are), whereas bisection points above 2,000 ms reflect duration underestimation (i.e., durations are perceived shorter than they actually are). An increase of “longer” responses after the tool-use session, as compared to the pre-tool session, induces a decreased PSE, reflecting that durations are perceived as being longer with respect to before tool-use session. Conversely, an increase of “short” responses after the tool-use session induces an increased PSE, reflecting that durations are perceived shorter. Thus, the PSE allows us to observe whether tool-use sessions induced a temporal judgment bias toward either an underestimation or an overestimation of durations in near and far spaces.

### Statistical Analysis

The Kolmogorov–Smirnov test was conducted to verify whether or not our dataset was normally distributed. Since we did not obtain significant results (*p* > 0.05), we assumed the dataset was not significantly different from a normal distribution. First, to explore the effect of temporal duration on time estimation judgment of participants, an ANOVA was conducted on the percentage of “long” responses recorded with intervals (1,600, 1,800, 2,000, 2,200, and 2,400 ms) as a within-subject variable.

Second, to explore the effect of a tool-use training on the temporal task, an ANOVA was conducted on mean PSE value with Group (young and aging participants) as a between-subject variable, and Session (pre- and post-tool), Condition (near and far spaces), Orientation (left and right), and Transector (–0.2°; 0.0°; +0.2°) as within-subject variables. When necessary, *post hoc* analyses were conducted using Newman–Keuls’s correction. The magnitude of effect size was expressed by partial eta squared (η^2^_p_).

## Results of Experiment 1: Time Bisection Task

### The Effect of Time Interval

The variable *interval* was significant [*F*(4,140) = 470.2; *p* < 0.0001; η^2^_p_ = 0.93]: *post hoc* analyses revealed that all temporal durations differ each other, revealing an increase of percentage of long response according to the increase of temporal durations (1,600 = 15%; 1,800 = 33%; 2,000 = 55%; 2,200 = 75%; 2,400 = 86%; *p* < 0.0001 for all comparisons; as shown in Figure 2B).

### The Effect of Muller-Lyer Illusion and Tool-Use on Temporal Estimation

The ANOVA conducted on the PSE revealed a significant effect for the variable *group* [*F*(1,34) = 3.19; *p* = 0.028; η^2^_p_ = 0.13]: an underestimation of temporal interval was found in elderly (mean ± SEM 2,019 ± 19.0) compared to younger participants (1,948 ± 17.7; see Figure 2C). Crucially, the interaction *Session* × *Condition* [*F*(1,34) = 5.69; *p* = 0.02; η^2^_p_ = 0.14] was significant. In the pre-tool session, temporal durations are perceived shorter (i.e., participants underestimated temporal interval) when stimuli were presented in far condition (2,075 ± 59.6) compared to near condition (1,954 ± 16.5; *p* = 0.045). In contrast, no such effect was observed in the post-tool session between near and far conditions (1,975 ± 27.9 vs. 1933 ± 21.7; *p* = 0.66). Interestingly, when pre- and post-tool sessions were directly compared, a significant reduction of time underestimation emerged comparing far conditions (*p* = 0.03), whereas no significant differences emerged comparing near conditions (*p* = 0.66; as shown in Figure 2D). No significant interaction for the factor Group and other variables was found (all *p*-values > 0.05).

### Interim Discussion of Experiment 1

First, time intervals were classified consistently with their effective durations, since the percentage of long response increased as the time interval increased. Second, age influences time estimation: elderly participants perceived temporal durations as shorter compared to young participants. Third, considering the depth dimension along the sagittal axis, our results highlight how temporal estimation is modulated by the space where the temporal stimulus is presented: temporal durations presented in far space are perceived shorter as compared to those presented in near space. This dissociation disappears following a long tool-use session, in which participants actively perform a series of consecutive movements to reach objects located in a far and unreachable space by means of a long rake. Indeed, after tool-use training, temporal stimuli presented in far space are processed as if they occurred in near space, demonstrating the role of action in the “remapping of time.” Crucially, after an equivalent tool-use training but with a short rake, this effect was not observed (refer to Supplementary Section Experiment 3). Indeed, temporal durations presented in far space are perceived shorter as compared to those presented in near space in the pre-training session, and this dissociation was still present after the short tool-use training (see Supplementary Figure 1). Finally, we found that the Muller-Lyer illusion, which alters the left/right coding, did not affect temporal estimation. This last result can be explained in two different ways. First, the near/far space, coded by action possibilities, is more relevant for time estimation than the left/right spatial bias induced by visual illusion. Second, participants could have not experienced the Muller-Lyer illusion at all. To rule out this last possibility, we assessed the occurrence of Muller-Lyer’s illusion by using a spatial bisection task.

## Experiment 2: Spatial Bisection Task

### Stimuli

**FIGURE 3:**
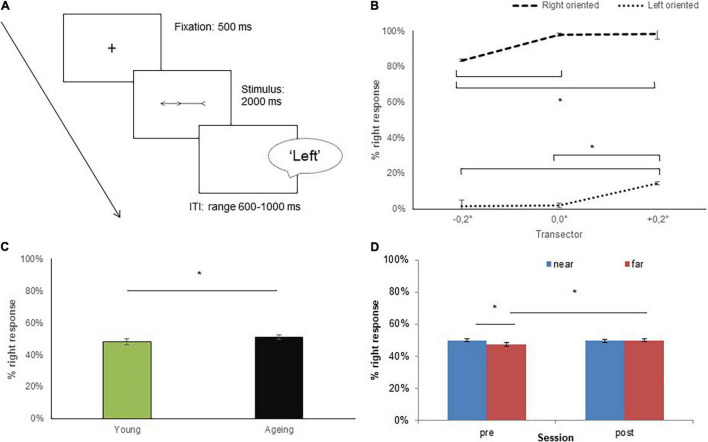
The experimental paradigm and performance of participants at the spatial bisection task. In the spatial bisection task, **(A)** participants had to verbally classify each stimulus as “left” if the transector was perceived as being closer to the left end of the line, and as “right” if they perceived it to be closer to the right end. **(B)** The percentage of right response expressed as a function of Muller-Lyer illusion. **(C)** The midpoint values are expressed as a percentage (%) of the right response as a function of Group. **(D)** The percentage of right response expressed as a function of conditions (near and far) and the tool-use session (pre and post). Error bars indicate SEM. Asterisks indicate significant differences.

We used the same stimuli of Experiment 1 (see Figure 1) but stimuli duration was fixed at 2,000 ms. According to the two illusory conditions adopted (left and right expanded) and the three transector positions (–0.2°; 0.0° +0.2°), six different prebisected lines were obtained. Each stimulus was presented six times in a random order, yielding a total of 72 trials (36 in the near space and 36 in the far space). Each trial began with the presentation of a fixation cross lasting 500 ms, then the stimulus was displayed for 2,000 ms and replaced by a white background, which remained visible until a response was made (as shown in Figure 3A).

### Procedure

The spatial bisection task was performed in near and far spaces and presented in separate blocks, counterbalanced across participants. The task was repeated two times, in the same order, before and after a tool-use session.

We adopted a left/right alternative forced-choice paradigm in which participants have to judge which end of the line the transector was closer to Weidner and Fink (2007). On each trial, participants were asked to verbally classify each prebisected line as “left” if the transector was perceived as being closer to the left end of the line, and as “right” if they perceived it to be closer to the right end.

A practice session was administered before the experimental task. In this practice session, participants had to classify four prebisected lines at –0.2° as “left,” and four prebisected lines at + 0.2° as “right,” all randomly presented. Participants received feedback only in these eight practice trials, which was repeated until they had reached at least 80% of accuracy (mean ± SD accuracy = 90.2% ± SD 0.05). Then, they were involved in the experimental task in which all the possible six prebisected lines were randomly displayed. Participants were instructed to make their best guess if they were unsure and to respond as accurately and as quickly as possible. No feedback was given in the experimental blocks.

The experimenter seated behind the participants (at least 1.5 m) and recorded the verbal responses of participants by pressing one of the two keys on a wireless mouse (left/right). Each block of the spatial bisection task took approximately 5 min to complete.

## Data Analysis

For each participant, to obtain an objective measure of the perceived line midpoint, we calculated the proportion of “right” responses (i.e., when subjects judged the transector as being at the right of the true center) in each of the four conditions (i.e., pre-tool-use/near space, pre-tool-use/far space, post-tool-use/near space, and post-tool-use/far space). As expected in line with the visual illusion, participants typically fail to correctly judge the midpoint of the line because half of the line appears shorter or longer than the real one with the ingoing fins (> <) and outgoing fins (< >), respectively (Ro and Rafal, 1996; Vallar et al., 2000; Daini et al., 2001).

An increase of “right” responses after the tool-use session, as compared to the pre-tool session, induces a relative shift toward the left of the perceived midline. On the contrary, an increase of “left” responses after the tool-use session induces a relative shift to the right.

## Results of Experiment 2: Spatial Bisection Task

### The Effect of Muller-Lyer Illusion and Tool-Use on Spatial Estimation

To explore the effect of a tool-use training on the spatial task, an ANOVA was conducted on the percentage of “right” responses recorded in each condition with Group (young and aging participants) as a between-subject variable, and Session (pre- and post-tool), Condition (near and far spaces), Orientation (left and right), and Transector (–0.2°; 0.0°; +0.2°) as within-subject variables.

The analysis revealed a significant effect for the variable *Group* [*F*(1,34) = 6.55; *p* = 0.02; η^2^_p_ = 0.16]: younger participants perceived the transector as closer to the left end of the line (mean ± SEM 48% ± 0.018) compared to the elderly adults (51% ± 0.013; see Figure 3C). Furthermore, confirming the effect of Muller-Lyer illusion, the interaction *Orientation* × *Transector* [*F*(2,70) = 10.9; *p* < 0.0001; η^2^_p_ = 0.23] was also significant. *Post hoc* comparisons revealed that for right-side- and left-side-oriented stimuli, the difference in judging the transector position was at 0.0°. Participants judged the transector as closer to the right end of the line when it was at +0.2° (98% ± 0.009) and 0.0° (98% ± 0.012) compared to –0.2° (84% ± 0.036; *p* < 0.0001). Conversely, for left-side-oriented stimuli, an increase in right responses was found when the transector was at +0.2° (14% ± 0.028) compared to 0.0° (2% ± 0.008; *p* < 0.0001) and at –0.2° (2% ± 0.008; *p* < 0.0001). Both these significant effects confirmed that responses of participants were affected by the direction of the illusion: right-side-oriented lines induce the opposite effect that is a shift of the midpoint toward the right, whereas left-side-oriented lines induce a shift of the midpoint (transector) toward the left (as shown in Figure 3B).

Crucially, the interaction *Session* × *Condition* [*F*(1,34) = 3.98; *p* = 0.05; η^2^_p_ = 0.10] was significant. In the *pre-tool session*, an increase in right response was found in near condition (50% ± 0.006) compared to far condition (48% ± 0.009; *p* = 0.048). In contrast, no such effect was observed in the *post-tool session* (near space = 50% ± 0.007 vs. far space = 50% ± 0.008; *p* = 0.89). More relevant for the purpose of this study, when pre- and post-training sessions were directly compared, no differences emerged when near conditions were considered (*p* = 0.91), whereas following the tool-use session, a significant increase in right response was found in far conditions (*p* = 0.046; as shown in Figure 3D). No significant interaction for the factor Group and other variables was found (all *p*-values > 0.05).

### Interim Discussion of Spatial Bisection Task

Results of the spatial bisection task put in evidence the effect of Muller-Lyer illusion on the perception of lines midpoint. In fact, *right-oriented stimulus* results in a shift of the midpoint toward the right. In contrast, *left-oriented stimulus* results in a shift of the midpoint toward the left. This result perfectly fits with the well-known effects of the Muller-Lyer illusion (Restle and Decker, 1977; Weidner and Fink, 2007). Moreover, age influenced the strength of the illusion: elderly participants showed a stronger rightward shift in the perceived line midpoint as compared to young participants. More interestingly for the current study, considering the depth dimension along the sagittal axis, our results demonstrate that the spatial judgment is also influenced by the stimulus position in near or far space: a leftward shift in the perceived line midpoint emerged in far as compared to near space. Furthermore, after a long tool-training, this dissociation disappears: when participants judged the midpoint of the line, no difference was found between stimuli presented in near and far spaces. Importantly, this effect was not found after training with a short tool (as shown in Supplementary Section Experiment 4). Indeed, the leftward shift in the perceived line midpoint reported in far as compared to near space was still present before and after the short tool-use training (see Supplementary Figure 2). Thus, the lack of an effect of the Muller-Lyer illusion on time perception in Experiment 1 (time bisection task) is not explained by the fact that participants did not perceive the illusion. Rather, these results suggest that time is spatially organized, specifically when the space is encoded as a “working space” on which to act.

## General Discussion

The main purpose of this study was to explore the interaction between left/right and near/far spatial coding in temporal estimation and their functional relationship. To address this, in the time bisection task, stimuli were manipulated both along the transversal left-right axis, according to the Muller-Lyer illusion, and along the sagittal axis, by presenting stimuli in reachable/near and unreachable/far space.

The first relevant result is the influence of near/far spatial coding on temporal estimation. Before the tool-use training, there was a clear dissociation between near and far spaces: temporal durations are perceived shorter when stimuli are presented in far as compared to near space. To explain this finding, we hypothesize that the temporal duration of stimuli presented close to the body (i.e., near space) was perceived as slower and prolonged because of the influence of action preparation on time perception. Curiously, experts in motor performance, such as tennis and football players, usually report the feeling of the ball “slowing-down” just before hitting it and comment that they “see” the ball more clearly before striking it (Murphy and White, 1978; Hagura et al., 2012). This implies that the subjective passage of time may be influenced by action, especially when participants performed movements close to their bodies (De Kock et al., 2021). Supporting this evidence, in a temporal judgment task, Hagura et al. (2012) found that stimuli were significantly overestimated (i.e., higher PSE values) when participants prepared for action compared with a control condition in which they had to detect visual stimuli. These results confirmed the claim that during motor preparation, participants perceived the visual stimulus to have a longer duration. Then, a possible interpretation is that to accurately perform movements, the optimal strategy for the brain is to enhance the detection of any environmental changes before motor execution and that this leads to a perception of slowing-down time. In addition, the space near the body is also a multisensory space in which sensory inputs that are spatially and temporally close are likely to be bound (Meredith et al., 1987; Stein and Stanford, 2008). Considering that, recent evidence demonstrated that the temporal binding window, i.e., the temporal window which is more likely that different sensory stimuli are integrated and perceived as synchronous, is larger in the near than in the far space (Noel et al., 2016). Indeed, in a simultaneity judgment task, when multisensory stimuli were presented close to the body, individuals judged sensory inputs as co-occurring over a wide range of temporal intervals. A larger temporal binding window in the near space can be responsible for slowing down the temporal perception observed near the body. This, in turn, can provide a more efficient working space for the body to act in time on nearby objects.

Interestingly, we demonstrated that tool-use training eliminates the dissociation between near and far spaces in time estimation, reporting the so-called *remapping of time*. This finding is in line with a previous study on young healthy participants (Anelli et al., 2015) and corroborates the idea that time is a physical dimension that interacts with the reachability and action potentialities of an individual in space.

Supporting this hypothesis, a similar dissociation between near and far spaces, and a remap of far and near spaces after the long tool-use training, were also found in the spatial bisection task. Taken together, our findings converged to the idea that temporal and spatial representations dynamically change according to the action potentialities of an individual. Coherently with this interpretation, following the short tool-use training, no changes were observed either when temporal or spatial judgments were provided in time and spatial bisection tasks, respectively. This evidence is in line with several studies, demonstrating that a short tool-use does not expand the action capability of an individual (Farnè et al., 2005; Costantini et al., 2011; Bourgeois et al., 2014; Anelli et al., 2015; Patané et al., 2016b; Candini et al., 2019). The second finding is that the Muller-Lyer illusion, which changes the perception of stimulus extension along the left/right dimension, does not influence time estimation. This is surprising if we consider the numerous evidence showing an underestimation of left stimuli duration compared to the right ones (Vallesi et al., 2008; Vicario et al., 2008; Frassinetti et al., 2009). However, in previous studies, such link between space and time emerged when the spatial position of stimuli was prioritized (Magnani et al., 2012) or when responses were lateralized by using a motor response code (Torralbo et al., 2006; Santiago et al., 2007; Ishihara et al., 2008; Vallesi et al., 2008; Anelli et al., 2016, 2018). Moreover, left/short and right/long association was demonstrated when a left or right shift of visuospatial attention was induced through the prismatic adaptation procedure (Frassinetti et al., 2009; Magnani et al., 2011; Oliveri et al., 2013; see for a review Anelli and Frassinetti, 2019). Thus, the Muller-Lyer illusion does not affect time estimation, probably because it is based on visual perception rather than on spatial attention. The perceptual nature of this illusion is supported by neuroimaging studies, showing that activity in the occipital extrastriate cortex, especially the lateral occipital cortex, correlates with the strength of the Muller-Lyer illusion (Weidner and Fink, 2007; Plewan et al., 2012). Neuropsychological evidence on patients affected by stroke, which is one of the leading causes of disability (Scrutinio et al., 2020), also indicates that the lack of the illusion correlates with the degree of damage in occipital regions in patients with the visual deficit, i.e., hemianopia (Vallar et al., 2000; Daini et al., 2001; Vuilleumier et al., 2001). Therefore, one of the novelties of our results is that time perception is not modulated by a pure perceptual visual illusion that does not affect visual spatial attention. A further critical result for this study concerns the role of age on time and space processing. In the temporal bisection task, young participants perceived temporal duration as longer compared to elderly participants, and in the spatial bisection task, they showed a leftward bias. Coherent with the hypothesis, that time is spatially represented, this bias due to a leftward shift of spatial attention (Friedrich et al., 2018) may influence time perception, inducing a shorter reference-time-interval stored in memory in young than in elderly participants. Consequently, when young people compare interval duration with the reference time in memory, they overestimate the duration of stimuli. In contrast, in elderly people, who do not show the leftward bias, the reference-time-interval stored in memory is longer than in young, and then they underestimate the stimuli duration. These data could suggest that individual differences in spatial attention may play a role in the construction of the memory temporal standard by which durations are categorized.

Our results are in line with previous works on temporal processing which demonstrated that the underestimation bias found in temporal judgment in elderly people is due to a deceleration of the internal clock speed (Craik and Hay, 1999; Perbal et al., 2002). For instance, Craik and Hay (1999) argued that the pacemaker of elderly participants emits lower rate pulses that contribute to slowing down of the internal clock. This interpretation well fits with the common feeling that the sense of the passage of time is fast in aging.

## Conclusion

Our results provide further support to the existence of a close relationship between time and space which is mainly driven by the closeness of the stimulus to the body. This finding reveals the highly flexible and plastic nature of time representation which could be for humans an optimal strategy to enhance detection of sensory information in the environment to accurately tune both spatial and temporal information during everyday activities.

## Data Availability Statement

The raw data supporting the conclusions of this article will be made available by the authors, without undue reservation.

## Ethics Statement

The studies involving human participants were reviewed and approved by University of Bologna. The participants provided their written informed consent to participate in this study.

## Author Contributions

MC and FF designed this study. MC collected and analyzed the data and wrote this manuscript. MD’A and FF provided revisions. All authors approved the final version of this manuscript for submission.

## Conflict of Interest

The authors declare that the research was conducted in the absence of any commercial or financial relationships that could be construed as a potential conflict of interest.

## Publisher’s Note

All claims expressed in this article are solely those of the authors and do not necessarily represent those of their affiliated organizations, or those of the publisher, the editors and the reviewers. Any product that may be evaluated in this article, or claim that may be made by its manufacturer, is not guaranteed or endorsed by the publisher.
