# Supplementary material for: Time Interaction With Two Spatial Dimensions: From Left/Right to Near/Far
Source: Front Hum Neurosci. 2022 Jan 18;15:796799. doi: 10.3389/fnhum.2021.796799 (PMC8804530; doi:10.3389/fnhum.2021.796799)
Supplement: Supplementary file 1 [file Data_Sheet_1.docx]

**Supplemental information**

We conducted two further experiments to demonstrate that the effects of tool use on the Temporal Bisection (Experiment 1) and Spatial Bisection tasks (Experiment 2) were related to an extension of the reaching space. Eight-teen new healthy adults performed the Temporal Bisection task (Experiment 3) and Spatial Bisection task (Experiment 4) before and after a tool-use training with a short rake. The procedure was exactly the same as in Experiments 1 and 2, except that in the tool-use session participants used a *short* tool (10 cm) to reach the poker fiches placed within the arm’s reaching space.

*Short tool-use session*

During the tool-use training, the experimenter randomly placed on the table a series of colored poker chips at a distance of ≈30 cm from the participant’s trunk (i.e., inside the reaching space). The chips were presented in the participants’ midsagittal axis (0°), or at 10° and 20° to the left and to the right of the central position (-10°, -20°, +10°, +20°). The participant performed about a total of 100 reaching movements in order to bring the objects close to their body with no time constraint by using a 10 cm-long rake.

**Experiment 3: Time Bisection task before and after a short tool-use session**

*Participants*

Eight-teen right-handed neurologically healthy volunteers with normal or corrected-to-normal vision were recruited (9 males, mean ± sd age = 27.3 ± 8.2 years; mean ± sd education = 15.4 ± 2.9 years). All participants were naive to the purpose of the research and provided written informed consent to participate in the study.

*Statistical analysis*

To study the effect of temporal duration on participant’s time estimation judgment, an analysis of variance (ANOVA) was conducted on the percentage of “long” responses recorded with Interval (1600, 1800, 2000, 2200, 2400 ms) as within-subject variable.

Then, to verify the effect of a short tool-use training on temporal estimation, an ANOVA was conducted on mean PSE value with Session (Pre- and Post-tool) and Condition (Near and Far space) as within-subject variables.
When necessary, post-hoc analyses were conducted by using the Newman-Keuls’s correction. The magnitude of effect size was expressed by partial eta squared (η^2^_p_).

*Results of Experiment 3*

The variable **Interval** was significant [F(4,68)=307.4; p<.0001; η^2^_p_=.95]: post hoc analyses revealed that all temporal durations differ each other revealing an increase of percentage of long response according to the increase of temporal durations (1600=10%; 1800=28%; 2000=54%; 2200=75%; 2400=88%; p<.0001 for all comparisons; see Figure S1a).

The ANOVA conducted on the PSE revealed a significant effect for the variable **Condition** [F(1,17)=9.94; p=0.006; η^2^_p_ =.37]: temporal durations are perceived shorter (i.e., participants underestimated temporal interval) when stimuli were presented in far (2250±528.1) compared to near condition (1983±184.2; see Figure S1b). Importantly, the interaction Session x Condition was not significant [F(1,17)=0.13; p=0.73; η^2^_p_ =.01] suggesting that this dissociation was present either in the pre-tool session (far: 2276 vs. near: 1977) or post-tool session (far: 2223 vs. near: 1988; see Figure S1c).


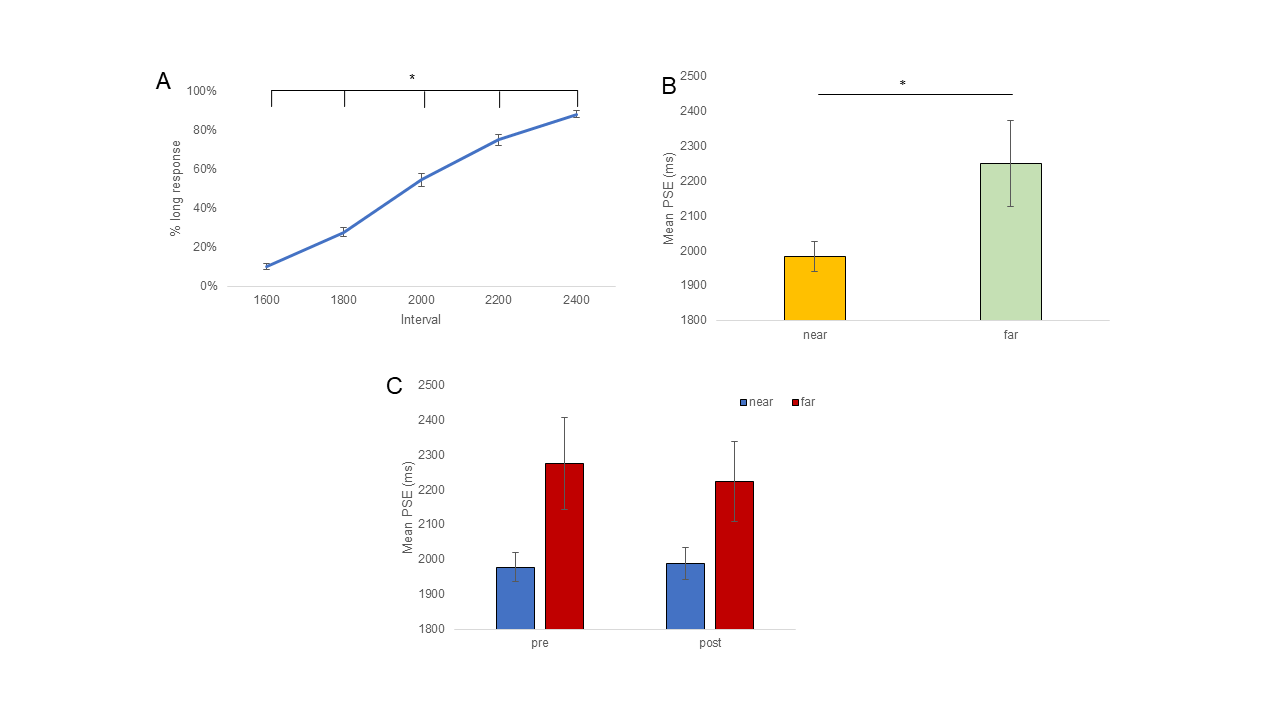


Figure S1. **Participants’ performance at Time Bisection task**. The percentage of long response as a function of duration interval (a). The point of subjective equality judgments (PSE) expressed as a function of Condition (near and far) (b) and Condition x Session (pre and post) (c). Error bars indicate standard error of means (SEM). Asterisks indicate significant differences.

**Experiment 4: Spatial bisection task before and after a short tool-use session**

The same group of participants were submitted to the Spatial bisection task before and after a short tool-use session.

*Statistical analysis*

To explore the effect of a short tool-use training on spatial estimation, the same ANOVA was also conducted on the percentage of right response recorded in each condition with Session (Pre- and Post-tool) and Condition (Near and Far space) as within-subject variables.
When necessary, post-hoc analyses were conducted by using the Newman-Keuls’s correction. The magnitude of effect size was expressed by partial eta squared (η^2^_p_).

*Results of Experiment 4*

The analysis revealed a significant effect for the variable **Condition** [F(1,17)=8.68; p=0.009; η^2^_p_ =.34]: an increase in right response was found in near (50%±0.004) compared to far condition (49%±0.004; p=.048; see Figure S2a). Crucially, no significant interaction Session x Condition was found [F(1,17)=0.62; p=0.44; η^2^_p_ =.03] suggesting that this dissociation was present either in the pre-tool session (near: 51% vs. far: 49%) vs. or post-tool session (near: 50% vs. far: 48%; see Figure S2b).


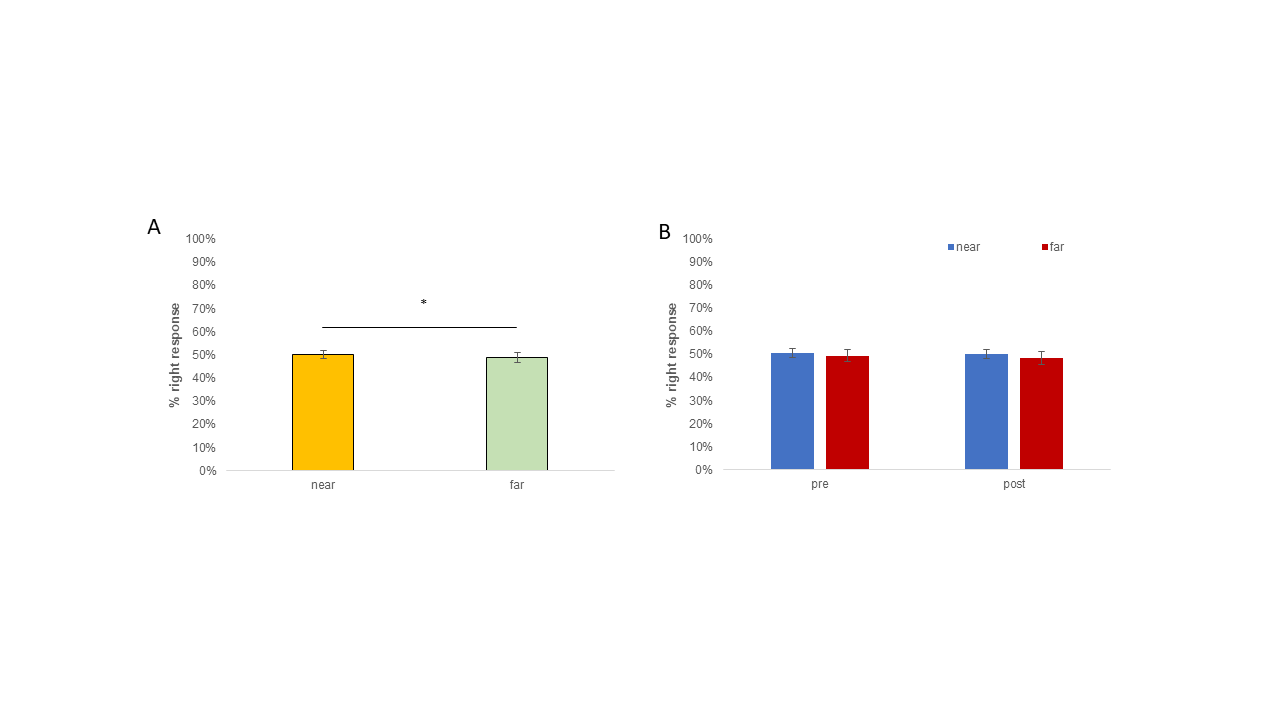


Figure S2. **Participants’ performance at Spatial Bisection task**. Midpoint values expressed as percentage (%) of right response as a function of conditions (near and far) (a) and Condition x Session (pre and post) (b). Error bars indicate SEM. Asterisks indicate significant differences.
